# Testicular cancer incidence and associations with prior epididymo-orchitis or urinary tract infections: a national cohort study in Sweden, 1964–2018

**DOI:** 10.1136/bmjonc-2026-001078

**Published:** 2026-04-29

**Authors:** Filip Jansåker, Xinjun Li, Kristina Sundquist

**Affiliations:** 1 Center for Primary Health Care Research, Department of Clinical Sciences Malmö, Lund University, Malmö, Sweden; 2 University Clinic Primary Care, Skåne University Hospital, Region Skåne, Malmö, Sweden; 3 Department of Clinical Microbiology, Rigshospitalet, Copenhagen University Hospital, Copenhagen, Denmark; 4 Center for Community-based Healthcare Research and Education (CoHRE), Department of Functional Pathology, School of Medicine, Shimane University, Shimane, Japan; 5 Department of Family and Community Medicine, McGovern Medical School, The University of Texas Health Science Center, Houston, Texas, USA

**Keywords:** Epidemiology, Testicular cancer

## Abstract

**Objective:**

To examine the extent to which epididymo-orchitis or urinary tract infections (UTI) may precede testicular cancer (TC) in Sweden.

**Methods and analysis:**

We conducted a nationwide, open-cohort study including 8 382 433 men between 1964 and 2018. Standardised incidence ratios (SIR) with 95% CIs were calculated to compare TC incidence rates in men diagnosed with epididymo-orchitis or UTI—either in the same calendar year as TC or in preceding calendar years (mean follow-up: 6.85 years, ±8.31 SD)—with those in men without these infections. Analyses were controlled for potential confounders and a sensitivity analysis on cystitis was also conducted.

**Results:**

A total of 11 903 men were diagnosed with TC during the study period; of these, 400 (3.36%) had been diagnosed with epididymo-orchitis and 122 (1.02%) with UTI. The TC incidence rate per 100 000 person-years was 5.09 (95% CI 4.99 to 5.18) for the entire study period and increased from 2.84 (2.68 to 3.02) in 1964–1973 to 8.37 (7.99 to 8.77) in 2014–2018. Among men with epididymo-orchitis (n=89 596), TC was diagnosed in 0.45%, with an overall SIR of 6.34 (95% CI 5.73 to 7.00) in the full model. For TC diagnosed in the same calendar year as epididymo-orchitis (289 cases), the SIR was 85.97 (76.33 to 96.50). For TC diagnosed in subsequent calendar years (111 cases) the SIR was 1.86 (1.53 to 2.24), with most cases diagnosed within 1–4 calendar years of follow-up (65 cases). Among men with UTI (n=294 201), TC was diagnosed in only 0.04%, with an overall SIR of 1.74 (1.44 to 2.08). The associations were not significant for TC diagnosed 1–4 years after UTI, nor between cystitis and subsequent TC.

**Conclusion:**

The association between epididymo-orchitis and TC was strong and persisted for several years after the infection. The findings support clinicians maintaining a heightened awareness of TC in patients with epididymo-orchitis, particularly when in diagnostic doubt or if symptoms persist, and could be a foundation for more detailed clinical studies on epididymo-orchitis as a potential TC risk factor. Although a potential link between UTI and TC was identified, the absolute risk was almost negligible.

**WHAT IS ALREADY KNOWN ON THIS TOPIC**

- Epididymo-orchitis and urinary tract infections (UTI) may precede testicular cancer (TC) diagnosis, but potential associations in large population-based studies have not been examined.

**WHAT THIS STUDY ADDS**

- In this Swedish nationwide study, we found that epididymo-orchitis and UTI may precede TC diagnosis.
- The associations between epididymo-orchitis and TC were strong and persisted for several years after the infection, whereas the association between UTI and TC was weaker and no association was observed between cystitis and TC.
- Approximately 0.45% of men with epididymo-orchitis and 0.04% of men with UTI were diagnosed with TC in the same or subsequent calendar years.

**HOW THIS STUDY MIGHT AFFECT RESEARCH, PRACTICE OR POLICY**

- The findings may be useful information in clinical practice and could provide a foundation for further clinical research.

## Background

 Testicular cancer (TC) is the leading cancer in young men aged 15–40 years, especially those of European origin, with increasing incidence rates worldwide.^1^,^5^ Incidence rates start to rise in late adolescence, spike at around 30 years of age and then rapidly decline.^6^ TCs are predominantly germ cell tumours (seminomas and non-seminomas), which account for 90%–95% of all cases, with non-seminomas dominating in young ages and seminomas in older ages.^3^,^57^ Early diagnosis is crucial, as TC is highly curable at an early stage by radical orchiectomy,^4 7^ and delayed diagnosis correlates with poorer survival.^8^ High awareness of TC symptoms and misdiagnosis-prone conditions, as well as knowledge of potential clinical markers and risk factors for TC, is therefore important.

Possible risk factors for TC include cryptorchidism, genetic factors, familial TC, maternal smoking, certain occupations and viral infections.^49^,^13^ Earlier studies also found that sexually transmitted infections and childhood mumps orchitis were associated with TC,^11 12 14^ although more recent findings have been inconsistent.^15^ In clinical practice, the classical presentation of TC is a painless testicular lump, scrotal swelling and enlargement.^4 16^ However, some men may present with other symptoms and/or be diagnosed or misdiagnosed with other conditions, such as infections, before TC diagnosis, which may delay detection.^716^,^19^ For example, undiagnosed TC may present with acute scrotal pain, swelling and enlargement (eg, due to intratumoral haemorrhage or infarction), clinically mimicking epididymo-orchitis and resulting in misdiagnosis.^16 18 19^ A case–control study also found that testicular or groin pain strongly predicted TC and that 11% of men who visited primary healthcare in the year before TC diagnosis were diagnosed with epididymo-orchitis^16^—an association consistent with a prior study showing a strong link between TC and preceding epididymo-orchitis.^20^

Epididymo-orchitis is a common inflammatory disease of the epididymis and testis,^17 21^ often occurring at the same age as TC incidence peaks.^6^ Although isolated orchitis can occur due to a viral infection (eg, mumps orchitis), it more commonly occurs with concurrent epididymitis (epididymo-orchitis). The typical clinical presentation is a gradual onset of unilateral scrotal pain, enlargement and swelling (overlapping with TC symptoms), often accompanied by symptoms of urinary tract infection (UTI).^17^ The condition is mainly caused by sexually transmitted infections or enteric bacteria, usually Enterobacteriaceae, that also cause UTI.^22^,^17 21^ To our knowledge, no large-scale population-based study with long-term follow-up has examined the extent to which epididymo-orchitis or UTI may precede TC diagnosis. Prior research has primarily been case–control studies on isolated orchitis,^15^ and a recent review concluded that the available evidence for an association between epididymo-orchitis and TC is low.^23^ For UTI, only one previous study has investigated the potential link with TC, which likely was underpowered to detect any potential association.^15^

Sweden is an ideal setting for addressing these gaps, due to its universal tax-financed healthcare system^24^ and comprehensive national registers with long-term coverage—including a national cancer register^25^ and population-based data derived from primary healthcare settings, where most infections are diagnosed. We have recently identified potential short- and long-term associations between UTI and prostate- and urinary tract cancers in men using these data.^26^ In this study, we aimed to examine potential associations between epididymo-orchitis or UTI and TC diagnosis during 1964–2018.

## Material and methods

### Design, setting and study population

We conducted a nationwide, open-cohort study including 8 382 433 male individuals residing in Sweden during 1964–2018. Sweden has a universal tax-financed healthcare service with the goal of providing healthcare on equal terms for the entire population.^24^ At birth or on immigration, all individuals residing in Sweden are assigned a unique 10-digit personal identification number.^27^ This number is used in all healthcare contacts and for the collection of data to national registers by the public authorities, thus enabling accurate linkage between public registries and medical data. For this study, data linkages were performed by using a pseudonymized version of this number.

### Data sources

The Swedish National Cancer Register, which is managed by the Swedish National Board of Health and Welfare (in Swedish: Socialstyrelsen), is used for monitoring cancer incidence and survival rates in the country.^25^ Approximately 60 000 unique cancer diagnoses have been reported to this register annually, given a date of clinical diagnosis and registered by diagnosis (topography) codes and tumour classification (histology and morphology) codes. To ensure consistency, these codes were transferred to the 7th edition International Classification of Diseases (ICD-7) codes and Pathological Anatomical Diagnosis (PAD) codes for the entire study period. The Swedish National Patient Register, which is managed by the Swedish National Board of Health and Welfare (in Swedish: Socialstyrelsen), includes inpatient and outpatient specialist care medical diagnoses.^28^ During the study period, diagnosis codes were collected according to the following ICDs: ICD-7 (1964–1968), ICD-8 (1969–1986), ICD-9 (1987–1996) and ICD-10 (1997–2018). Population-based primary healthcare data, which includes ICD-coded healthcare data from 20 out of 21 administrative healthcare regions in Sweden, was also used in the study.^29^ The population-based coverage of this data varied over time and region throughout the 29-year time period (1990–2018) the data were collected, covering around 90% of the Swedish population in 2018. The Total Population Register, which is managed by the Swedish governmental authority Statistics Sweden (SCB), includes data on the whole Swedish population, including death, emigration, immigration and sociodemographic factors.^30^ The Multi-Generation Register is managed by SCB and includes data on (biological) parental and sibling linkages in the Swedish population for individuals born 1932 and afterwards.^31^

### Testicular cancer

The outcome was the primary TC incidence during the study period. The ICD-7 diagnosis code ‘178’ (malignant neoplasm of testis) was used to identify outcome event, collected from the Swedish Cancer Register (online supplemental table S1). TCs with PAD code ‘066’ were classified as seminomas and ‘826’ as non-seminomas (including mixed cell types). Due to differences in age distribution (non-seminoma predominating at younger ages and seminoma at older ages) and temporal incidence trends (higher increase in non-seminomas than seminomas),^3^,^57^ we also analysed seminomas and non-seminomas separately. In these analyses, TCs with other PAD codes (2.6%) were included in the non-seminomas group. Secondary (metastatic) cancer to the testis or haematolymphoid neoplasms of the testis were not included.

### Epididymo-orchitis and UTI

The predictors were defined as epididymo-orchitis and UTI occurring in the same or preceding calendar years to any potential TC diagnosis. For epididymo-orchitis, the diagnosis codes (online supplemental table S1) used were ICD-10 code ‘N45’ (orchitis and epididymitis), ICD-9 and ICD-8 code ‘604’ (orchitis and epididymitis) and ICD-7 code ‘614’ (orchitis and epididymitis). For UTI, the diagnostic codes used were: ICD-10 codes ‘N30’ (cystitis) and ‘N39’ (other disorders of urinary system, including ‘N39.0’, UTI, site not specified), ICD-9 and ICD-8 codes ‘595’ (cystitis) and ‘599’ (other disorders of urethra and urinary tract, including ‘599.0’, unspecified UTI), and ICD-7 codes ‘605’ (cystitis) and ‘609’ (other diseases of urinary system/urethra). While ICD-10 codes have been validated for UTI,^32^,^34^ the older ICD-codes for UTI and the codes for epididymo-orchitis have, to our knowledge, not. Nevertheless, all diagnosis codes for cystitis and epididymo-orchitis were more or less specific for these conditions, thus making it unlikely that the codes were used for other clinical conditions. However, the other codes used to identify UTI also include other diseases of the urinary system. Given that we only had access to category-level codes (eg, ICD-9 ‘599’) and not subcategory-level codes (eg, ICD-9 ‘599.0’) for most of the study period, we conducted a sensitivity analysis stratified by UTI diagnosis codes specific for cystitis and other codes (in this paper called: UTI, unspecified/site not specified).

### Diagnostic methods for TC, epididymo-orchitis and UTI (1964–2018)

The gold standard for TC diagnosis is histopathological examination following orchidectomy.^35^ PAD codes indicating histopathological verification were registered for more or less all TC cases identified in the National Cancer Register during the study period (online supplemental figure S2). TC evaluation in clinical practice evolved during the study period, initially based on symptoms and physical examinations, with the gradual implementation of complementary diagnostic tools such as scrotal ultrasound. The European Association of Urology included scrotal ultrasound in the 2001 TC guidelines,^36^ but it was probably used in clinical practice in Sweden before that. For example, adding ultrasound to improve physical examination was mentioned in a Scandinavian review on TC from 1985.^37^ Diagnostic methods for male UTI and epididymo-orchitis also evolved during the study period. Diagnosis has mainly been based on symptoms and physical examinations, with gradual addition of complementary tools such as urine dipstick and cultures, as well as scrotal ultrasound for differential diagnosis of acute scrotal pain.^38^ Urine cultures appeared to have been routinely used in Sweden from the 1970s and onward (including in primary healthcare from the 1980s)^39^ and scrotal ultrasound for differential diagnosis of acute scrotal pain was mentioned in a Swedish publication in 1994.^40^ To partly address the potential effect of these changes in diagnostic practice during the study period, which may have had geographical variability in implementation, we included time period and region of residence as covariates in the analyses.

### Covariates

Covariates include the time period of TC diagnosis (1964–1973, 1974–1983, 1984–1993, 1994–2003, 2004–2013 and 2014–2018), sociodemographic factors and comorbidities. The sociodemographic factors included age at TC diagnosis (<20, 20–39, 40–59, 60–79 and ≥80 years of age); educational level (≤9, 10–12 and >12 years of formal education), defined as the highest level during the study period; region of residence (large cities (Malmö, Gothenburg and Stockholm), Southern and Northern Sweden); and country of origin (born in or outside of Sweden).^30^ Family history of TC was defined as a TC diagnosis in first-degree male relative (brother or father). The comorbidities included diagnosis of alcoholism, cannabis use, chronic obstructive pulmonary disease (COPD) as a proxy for heavy tobacco smoking, diabetes mellitus, HIV infection (HIV) and obesity during the study period (online supplemental table S1). These covariates were included in the analyses as potential confounders. Sensitivity analyses stratified by age at TC diagnosis were also conducted.

### Statistical analysis

Each male individual was followed from birth or immigration (starting on 1 January 1964) until the year of a first TC diagnosis, emigration, death or end of study period (31 December 2018), whichever came first. In those with epididymo-orchitis or UTI, each individual was followed from the year the infection (first event) was diagnosed (starting on 1 January 1964) until the year of a first TC diagnosis, emigration, death or end of study period, whichever came first. We calculated the total follow-up time in person-years, mean (SD) follow-up time and mean (SD) age at TC diagnosis. Incidence rates were calculated by combining data from the Total Population Register and Swedish Cancer Register. We calculated the distribution of the number of diagnoses and incidence rates (per 100 000 person-years) of TC in the total population during the study period. We also plotted TC incidence rates over the different time periods and in individuals with and without a preceding epididymo-orchitis or UTI by age at TC diagnosis. Standardised incidence ratios (SIRs) for TC were calculated to compare the observed TC incidence in individuals with epididymo-orchitis or UTI to the TC incidence in those without these infections, using indirect standardisation methods.^41^ All calculations were standardised by age groups, time period groups and other covariates. We categorised time periods of TC diagnosis as diagnosis recorded within the same or subsequent calendar years to the epididymo-orchitis or UTI diagnosis. Subsequent calendar years were also subcategorised into 1–4 and ≥5 years of follow-up. For epididymo-orchitis, the association with TC was tested in three models: model 1, adjusted for age and time period; model 2, adjusted for age, time period, sociodemographic factors and family history of TC; and model 3, a full model, adjusted for all covariates. SIRs were also calculated for different age groups to examine whether the relationships varied by age. In addition, we conducted a sensitivity analysis stratified by type of UTI diagnosis. The 95% CIs of the SIRs were calculated assuming a Poisson distribution. All of the analyses were performed using SAS software V.9.4 (SAS Institute).

## Results

The study population consisted of 8 382 433 male individuals, with a follow-up of 239 890 652 person-years and a mean (SD) follow-up of 27.93 years (±18.95; online supplemental table S3). A total of 11 903 individuals were diagnosed with TC, mainly seminomas (55%) and non-seminomas (42%) (online supplemental table S2), at a mean (SD) age of 36.9 (±13.5) years during the study period. Among these individuals, 522 (4.38%) had been diagnosed with epididymo-orchitis (n=400, 3.36%) or UTI (n=122, 1.02%) in the same or preceding calendar years (mean follow-up: 6.85 years, SD±8.31).

### TC incidence rates in Sweden between 1964 and 2018

**Figure 1:**
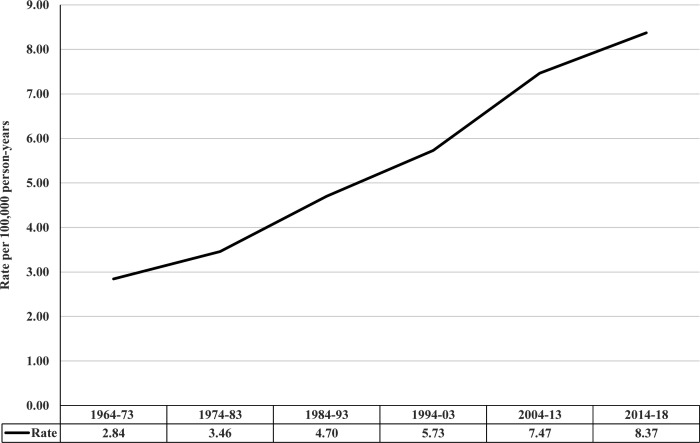
Testicular cancer incidence rates in Sweden between 1964 and 2018, age-standardised to the world standard population.

Table 1 shows that the TC incidence rate per 100 000 person-years was 5.09 (95% CI 4.99 to 5.18) during the entire study period, 11.17 (95% CI 10.92 to 11.43) in those aged 20–39 years, and increased from 2.84 (95% CI 2.68 to 3.02) in 1964–1973 to 8.37 (95% CI 7.99 to 8.77) in 2014–2018 (visualised in figure 1).

**Table 1:** Number of testicular cancer diagnoses and testicular cancer rates between 1964 and 2018 in Sweden

	Number of diagnoses		Rates per 100 000 person-years		
	n	%	Rate	95% CI	
Total population	11 903		5.09	4.99	5.18
Age at diagnosis (years)					
<20	447	3.76	0.75	0.68	0.82
20–39	7399	62.16	11.17	10.92	11.43
40–59	3201	26.89	5.13	4.96	5.32
60–79	750	6.30	1.74	1.62	1.87
≥80	106	0.89	1.28	1.06	1.55
Time period of diagnosis (years)					
1964–1973	1074	9.02	2.84	2.68	3.02
1974–1983	1431	12.02	3.46	3.28	3.64
1984–1993	1982	16.65	4.70	4.50	4.91
1994–2003	2423	20.36	5.73	5.50	5.96
2004–2013	3217	27.03	7.47	7.21	7.73
2014–2018	1776	14.92	8.37	7.99	8.77
Region of residence					
Large cities	5660	47.55	5.31	5.17	5.45
Southern Sweden	4565	38.35	4.92	4.78	5.06
Northern Sweden	1678	14.10	4.77	4.55	5.01
Educational level (years)					
≤9	3111	26.14	4.17	4.03	4.32
10–12	2642	22.20	4.62	4.45	4.80
>12	6150	51.67	5.96	5.81	6.11
Country of origin					
Born in Sweden	10 748	90.30	5.32	5.22	5.42
Born outside of Sweden	1155	9.70	3.62	3.42	3.84
Alcoholism					
Diagnosis	492	4.13	4.08	3.73	4.45
No diagnosis	11 411	95.87	5.14	5.05	5.24
Cannabis use					
Diagnosis	41	0.34	6.96	5.12	9.45
No diagnosis	11 862	99.66	5.08	4.99	5.17
COPD					
Diagnosis	478	4.02	4.77	4.36	5.21
No diagnosis	11 425	95.98	5.10	5.01	5.20
Diabetes mellitus					
Diagnosis	503	4.23	4.00	3.66	4.36
No diagnosis	11 400	95.77	5.14	5.04	5.23
HIV infection					
Diagnosis	19	0.16	7.85	5.01	12.31
No diagnosis	11 884	99.84	5.08	4.99	5.17
Obesity					
Diagnosis	103	0.87	4.78	3.94	5.80
No diagnosis	11 800	99.13	5.09	5.00	5.18
Family history of testicular cancer*					
Yes	198	1.66	31.70	27.58	36.44
Non	11 705	98.34	5.01	4.92	5.10

* Testicular cancer diagnosis in father or brother.

COPD, chronic obstructive pulmonary disease.

### TC incidence rates in relation to epididymo-orchitis and UTI

**Figure 2:**
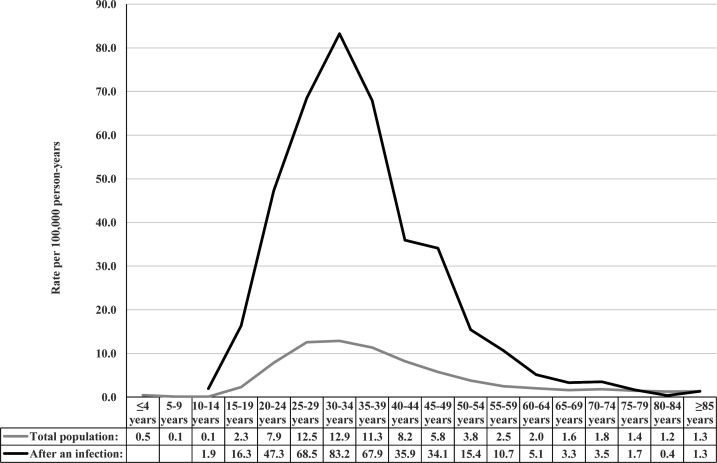
Testicular cancer incidence rates in the total Swedish male population (grey line) and in those with epididymo-orchitis or urinary tract infections (black line), 1964–2018, stratified by age at cancer diagnosis.

A total of 383 797 individuals were diagnosed with epididymo-orchitis (n=89 596) or UTIs (n=294 201) during the study period (online supplemental table S3). Among these individuals, the mean (SD) age at TC diagnosis was 37.1 years (±13.6), and the overall TC incidence rate was 28.21 (95% CI 25.89 to 30.74) per 100 000 person-years, corresponding to a total of 522 (0.14%) cases in those with epididymo-orchitis (n=400, 0.45%) or UTI (n=122, 0.04%). Figure 2 shows that the TC incidence rates appear to be higher among men with infections than those without until ≥75 years of age. The incidence rates were particularly high among men aged 25–39 years at TC diagnosis; peaking in those aged 30–34 years—in whom the rate was 83.2 and 12.9 cases per 100 000 person-years in those with and without epididymo-orchitis or UTI, respectively.

### Associations between epididymo-orchitis and TC

Table 2 shows that the associations between epididymo-orchitis and TC were consistent across the three models, with slight attenuations after adjustments. In model 1, the overall SIRs for TC associated with epididymo-orchitis compared with no infection were 6.57 (95% CI 5.94 to 7.25) for all TC, 6.13 for seminomas and 7.21 for non-seminomas. In model 3 (full model), the corresponding SIRs were 6.34 for all TC, 5.97 for seminomas and 6.88 for non-seminomas. For TC diagnosed in the same calendar year as epididymo-orchitis, the SIRs were exceptionally high, with SIRs of 85.97 for all TC, 80.54 for seminomas and 92.67 for non-seminomas in the full model. For TC diagnosed in subsequent calendar years to epididymo-orchitis, the associations decreased but remained significant, with an SIR of 1.86 (95% CI 1.53 to 2.24) for all TC in the full model. In the analysis stratified by follow-up time, the SIRs were 2.97 (95% CI 2.29 to 3.79) for all TC, 3.24 (95% CI 2.31 to 4.42) for seminomas and 2.62 (95% CI 1.69 to 3.87) for non-seminomas diagnosed 1–4 calendar years after epididymo-orchitis. Beyond this period, we were not able to detect any increased risk of TC following epididymo-orchitis.

**Table 2:** Standardised incidence ratios for testicular cancer diagnosis in men with epididymo-orchitis, by time period in relation to epididymo-orchitis diagnosis (1964–2018, Sweden)

Time period (calendar years)	O	Model 1			Model 2			Model 3		
		SIR	95% CI		SIR	95% CI		SIR	95% CI	
Seminomas										
The same year	150	**81.87**	**69.25**	**96.13**	**80.54**	**68.13**	**94.58**	**80.54**	**68.13**	**94.58**
Subsequent years	72	**2.10**	**1.65**	**2.65**	**2.03**	**1.59**	**2.56**	**2.05**	**1.60**	**2.58**
1–4 years	40	**3.29**	**2.35**	**4.49**	**3.22**	**2.30**	**4.39**	**3.24**	**2.31**	**4.42**
≥5 years	32	1.45	0.99	2.05	1.39	0.95	1.97	1.40	0.96	1.98
All	222	**6.13**	**5.35**	**7.00**	**5.93**	**5.18**	**6.77**	**5.97**	**5.21**	**6.81**
Non-seminomas										
The same year	139	**93.92**	**78.95**	**110.91**	**92.67**	**77.90**	**109.43**	**92.67**	**77.90**	**109.43**
Subsequent years	39	**1.68**	**1.19**	**2.30**	**1.62**	**1.15**	**2.22**	**1.60**	**1.14**	**2.19**
1–4 years	25	**2.69**	**1.74**	**3.97**	**2.63**	**1.70**	**3.88**	**2.62**	**1.69**	**3.87**
≥5 years	14	1.01	0.55	1.69	0.96	0.53	1.62	0.94	0.51	1.59
All	178	**7.21**	**6.19**	**8.35**	**6.97**	**5.99**	**8.08**	**6.88**	**5.90**	**7.96**
All testicular cancers										
The same year	289	**87.27**	**77.48**	**97.96**	**85.97**	**76.33**	**96.50**	**85.97**	**76.33**	**96.50**
Subsequent years	111	**1.93**	**1.59**	**2.33**	**1.87**	**1.54**	**2.25**	**1.86**	**1.53**	**2.24**
1–4 years	65	**3.03**	**2.34**	**3.87**	**2.96**	**2.29**	**3.78**	**2.97**	**2.29**	**3.79**
≥5 years	46	1.28	0.94	1.71	1.23	0.90	1.64	1.22	0.89	1.63
All	400	**6.57**	**5.94**	**7.25**	**6.36**	**5.75**	**7.01**	**6.34**	**5.73**	**7.00**

International Classification of Diseases (ICD) diagnosis codes for epididymo-orchitis: N45 (ICD-10), 604 (ICD-8, ICD-9) and 614 (ICD-7). ICD diagnosis code for testicular cancer: 178 (ICD-7). Model 1: adjusted for age and period; model 2: adjusted for age, period and sociodemographic factors (educational level, region of residence, country of origin) and family history of testicular cancer; model 3: adjusted age, period, sociodemographic factors, family history of testicular cancer and comorbidities (alcoholism, cannabis use, chronic obstructive pulmonary diseases, diabetes mellitus, HIV infection and obesity).

Bold values indicates that the 95% confidience interval does not include 1.00.

O, observations; SIR, standardised incidence ratio.

In the age-stratified analysis (online supplemental table S4), the SIRs for seminomas, non-seminomas and all TC were significantly elevated across all age groups with epididymo-orchitis compared with those without. The results also indicated stronger associations in individuals aged <20 years at TC diagnosis, with an SIR of 18.92 (95% CI 10.31 to 31.83) for all TC associated with epididymo-orchitis compared with no infection. However, only 14 TC cases were observed following epididymo-orchitis in this group, and most TC observations following epididymo-orchitis occurred in men aged 20–29 (n=105), 30–39 (n=153) and 40–49 (n=81) years at TC diagnosis. Among these groups, epididymo-orchitis was associated with an SIR for TC of 6.64 (95% CI 5.43 to 8.04) in men aged 20–29 years, 6.29 (95% CI 5.33 to 7.37) in men aged 30–39 years and 6.28 (95% CI 4.99 to 7.81) in men aged 40–49 years, compared with those in the same age groups without epididymo-orchitis.

### Associations between UTI and TC

Table 3 shows that the overall SIR for TC associated with UTI was 1.74 (95% CI 1.44 to 2.08) compared with no infection. The association was particularly strong for TC diagnosed in the same calendar year as UTI (SIR 8.79, 95% CI 6.01 to 12.42). It decreased considerably but remained significant for TC diagnosed in subsequent calendar years (SIR 1.35, 95% CI 1.08 to 1.66). In the age-stratified analysis (online supplemental table S5), the SIRs for TC associated with UTI were only significantly elevated in men aged 20–29 and 30–39 years at TC diagnosis compared with those without UTI in the same age groups. Moreover, the sensitivity analyses (online supplemental tables S5 and S6) show that there were few TC cases (19 observations) in men with cystitis, with no significant associations between cystitis and subsequent TC (14 observations). They also show that the association between UTI (unspecified/site not specified) and TC yielded largely similar results to the analyses including all UTI.

**Table 3:** Standardised incidence ratios for testicular cancer diagnosis in men with UTI, by time period in relation to UTI diagnosis (1964–2018, Sweden)

Follow-up times (years)	Urinary tract infection			
	O	SIR	95% CI	
Seminomas				
The same calendar year	20	**8.26**	**4.96**	**12.92**
Subsequent calendar years	52	**1.37**	**1.02**	**1.80**
1–4 years	21	**1.69**	**1.04**	**2.59**
≥5 years	31	1.22	0.83	1.73
All	72	**1.77**	**1.38**	**2.23**
Non-seminomas				
The same calendar year	13	**9.70**	**5.14**	**16.64**
Subsequent calendar years	37	1.31	0.92	1.81
1–4 years	3	0.40	0.08	1.18
≥5 years	33	**1.65**	**1.13**	**2.32**
All	50	**1.70**	**1.26**	**2.25**
All testicular cancers				
The same calendar year	33	**8.79**	**6.01**	**12.42**
Subsequent calendar years	89	**1.35**	**1.08**	**1.66**
1–4 years	24	1.20	0.77	1.79
≥5 years	65	**1.41**	**1.08**	**1.80**
All	122	**1.74**	**1.44**	**2.08**

Full model, adjusted for age, period and sociodemographic factors (educational level, region of residence and country of origin), family history of testicular cancer; and comorbidities (alcoholism, cannabis use, chronic obstructive pulmonary diseases, diabetes mellitus, HIV infection and obesity).

International Classification of Diseases (ICD) diagnosis codes for UTI were: N30 (ICD-10), 595 (ICD-8, ICD-9) and 605 (ICD-7) for cystitis and ICD diagnosis codes N39 (ICD-10), 599 (ICD-8, ICD-9) and 609 (ICD-7) for UTI, unspecified/site not specified. ICD diagnosis code for testicular cancer: 178 (ICD-7).

Cystitis is a lower urinary tract infection.

Bold values indicates that the 95% confidience interval does not include 1.00.

O, observations; SIR, standardised incidence ratio; UTI, urinary tract infection.

## Discussion

The main findings from this Swedish nationwide, open cohort study were that 522 (4.38%) of the 11 903 TC cases were diagnosed in the same calendar year as or in a subsequent year after a diagnosis of epididymo-orchitis (n=400, 3.36%) or potential UTI (n=122, 1.02%), with a mean follow-up of 6.85 years (SD±8.31). The association between epididymo-orchitis and TC was strong, with a 2.97-fold higher TC incidence within 1–4 years following the infection compared with no infection, and 0.45% of all individuals with epididymo-orchitis were diagnosed with TC. The association between UTI and TC was weaker and only 0.04% of those with UTI were diagnosed with TC, with no significant associations for all TC diagnosed 1–4 years after UTI, nor between cystitis and subsequent TC.

Similar to previous studies, we found that TC incidence rates increased over the study period,^1^,^3^ and spiked in men around 30 years of age, followed by a rapid decrease with increasing age.^6^ It has also been reported that men may present with symptoms of epididymo-orchitis before TC diagnosis.^7^ Potential associations between orchitis and TC were described several decades ago,^11 12^ but a meta-analysis from 2012 was only indicative of an association (pooled OR 1.80, 95% CI 0.74 to 4.42)—probably due to the few cases in the available studies (5–34 exposed cases).^15^ However, the authors also conducted an additional case–control study, including 767 TC cases (diagnosed between 2002 and 2005) matched to at least one control (n=929), in which they showed that an overall self-reported history of orchitis was associated with TC (OR 2.38, 95% CI 1.56 to 3.63). In that study, the authors also showed that the association was strong for orchitis occurring within one calendar year prior to TC diagnosis (OR 23.16, 95% CI 5.53 to 96.99), but beyond that period, they detected no significant association (OR 1.17, 95% CI 0.71 to 1.94). Potential associations between epididymo-orchitis and TC have been less studied^16 20^ and a recent review concluded that the available data were limited.^23^ One case–control study from Taiwan,^20^ based on health insurance data (2001–2013), found that men with TC (n=372) had a higher odds of epididymo-orchitis (OR 47.17, 95% CI 23.83 to 93.40) within 3 years before TC diagnosis compared with the 10:1 age-matched controls without TC, with 41 of the 53 epididymo-orchitis cases occurring in the TC group. Similarly, another case–control study from the UK^16^ found higher odds of preceding epididymo-orchitis diagnosis (OR 13, 95% CI 7.8 to 23.0) in men with TC compared with those without. This study was conducted between 2000 and 2012 and included men who had visited primary healthcare within 1 year before TC diagnosis, during which 153 (11%) of the men with TC (n=1398) and 37 (0.7%) of the matched controls (n=4956) were diagnosed with epididymo-orchitis.

The overall association between epididymo-orchitis and TC observed in our nationwide study (SIR: 6.34) was consistent with findings from these earlier case–control. We also observed that the association appeared strongest within the same calendar year, during which 289 (72.25%) of the 400 observed TC cases in men with epididymo-orchitis were diagnosed. Although we cannot determine the temporal sequence between TC and epididymo-orchitis diagnoses occurring within the same calendar year, the almost 86-fold increased TC incidence in those with epididymo-orchitis is likely not only explained by TC cases occurring prior to the infection. For example, in the case–control study from the UK,^16^ 153 (11%) of 1398 men with TC had been diagnosed with epididymo-orchitis in the preceding year, of whom 106 (71%) were diagnosed within 3 months before TC diagnosis. In addition, our study adds to the previous literature with findings showing that the association between epididymo-orchitis and TC persisted for 1–4 calendar years following the infection (SIR 2.97), but not beyond that period—although with an indication of higher TC (seminomas) incidence ≥5 years following epididymo-orchitis. The difference in design and larger sample size in our study probably explains why our study was able to detect significant associations between epididymo-orchitis and subsequent TC during longer follow-up, considering how few TC cases (n=111, 0.93%) were observed in subsequent calendar years to an epididymo-orchitis event in our nationwide study (11 903 TC cases).

We identified one earlier case–control study on TC and prior UTI, which showed no association between TC (n=767) and self-reported UTI (OR 0.96, 95% CI 0.69 to 1.33).^15^ In contrast, our nationwide study found an indication of a 1.74-fold increased TC incidence in men with UTI compared with men without. The association appeared particularly strong (SIR 8.79) within the same calendar year of diagnosis and remained elevated (SIR 1.34) in subsequent years. These findings may add to previous literature linking UTI to other forms of genitourinary cancers in men.^26 42 43^ However, only 0.04% of those with UTI were diagnosed with TC in our study, which could be regarded as a negligible absolute risk of TC following UTI, and there were too few TC observations (n=14) to detect any significant associations between cystitis and subsequent TC.

The mechanisms behind our findings probably vary depending on the timeframe of the associations. For TC diagnosed within the same calendar year as the infection, the association may partly be due to infections occurring subsequent to TC diagnosis, such as after urological procedures.^7 17 22 44 45^ However, other explanations should also be considered, such as misdiagnosis or reverse causation. For example, TC may initially be misdiagnosed as epididymo-orchitis,^18 19^ due to similar and overlapping symptoms (eg, scrotal pain, swelling and enlargement),^16^ and even sporadically misdiagnosed as UTI.^18^ It is also possible that TC may predispose the testis to infections by compromising its structure, with infections such as epididymo-orchitis being potential clinical TC markers. For TC diagnosed in subsequent calendar years, reversed causation or TC misdiagnosed as an infection is probably less likely, given that diagnostic delays for TC usually are measured in months.^8^ Instead, infections such as epididymo-orchitis might be potential risk factors for future TC, which may be explained by infection-related inflammation facilitating carcinogenesis,^46 47^ together with growing evidence of infections’ potential oncogenic effects on the human testis.^13^ However, potential causal links between infections and TC are complex, and our observational study cannot infer causality. The mechanisms underlying the observed associations therefore remain to be elucidated.

### Strengths and limitations

The study has several limitations that need to be considered. An important limitation is that we did not have data on symptoms or microbiological findings and could therefore not validate the epididymo-orchitis or potential UTI diagnoses. This limitation is particularly important to consider when interpreting the associations between UTI and TC, which were mainly attributed to diagnosis codes that include UTI as well as other diseases of the urinary tract system. Studies incorporating microbiological data are therefore needed to enhance the validity of the clinical diagnosis, especially for UTI. Another limitation is that men with urogenital infections may have been more likely to have TC detected due to scrotal palpation and/or subsequent diagnostic procedures, such as scrotal ultrasound. Any detection bias is probably most relevant for diagnoses occurring within the same calendar year, especially among patients with epididymo-orchitis, for whom scrotal palpation should be performed and scrotal ultrasound is recommended if diagnosis is uncertain or symptoms persist.^21^ Diagnostic bias could be particularly prevalent in years when scrotal ultrasound was used in clinical practice in Sweden. Adding scrotal ultrasound to improve physical examination was suggested in a Scandinavian review on TC in 1985,^37^ and for the differential diagnosis of acute scrotal pain in a Swedish publication from 1994.^40^ These changes in diagnostic practice may have resulted in a higher number of incidental TC findings in the years following the implementation of scrotal ultrasound in clinical practice.^48 49^ For example, a review indicated that 15% of TC cases are detected incidentally during acute scrotal ultrasound examinations.^48^ In addition, if some patients with infections were not evaluated for cancer during follow-up, we could have missed cancers in this group, but since this delay also would occur in those without infections, we expect this particular bias to be negligible. Misclassification of the infections may also have occurred, as the data from primary healthcare settings, where most infections are diagnosed, were not complete for the whole study period; however, this bias is likely non-differential. Additionally, milder infections or atypical presentations of epididymo-orchitis and UTI may have been missed and/or misdiagnosed (eg, as symptom diagnosis codes) and symptoms may also have been misdiagnosed as infections. Moreover, the infections, especially UTI, could be coincidental and unrelated to subsequent TC, which may result in artefactual associations, particularly during longer follow-ups. Although we adjusted for potential confounders, residual and unmeasured confounding may persist, but is probably low within the same calendar year of diagnosis, where reversed causation is expected. Lastly, our study is based on Swedish register data, which may limit its generalisability due to differences in healthcare systems and population demographics. Therefore, studies validating the associations in other countries and populations are warranted. The study also has several strengths that balance its limitations. First, the main strengths are the study size, long study period (55 years), and the use of several national registers of high quality.^25 28 30^ Additionally, data linkages were performed by using a pseudonymised version of the personal identification number, allowing for virtually complete coverage of Sweden’s population, inclusion of potential confounders, and practically no loss to follow-up.^27^ Moreover, the TC incidence rates in relation to age and time period (year) of diagnosis were consistent with prior studies,^1^,^36^ which support robustness and may indicate generalisability of our findings.

For clinical practice, most patients with epididymo-orchitis can likely be diagnosed and treated without further evaluations for TC, given that only 0.45% of patients with epididymo-orchitis were also diagnosed with TC in our study. Nevertheless, the strong association between epididymo-orchitis and TC within the same calendar year of diagnosis supports clinicians maintaining a heightened awareness of TC in patients presenting with epididymo-orchitis symptoms and during follow-up treatment of the infection. As recommended in the 2024 European guidelines on the management of epididymo-orchitis,^21^ scrotal ultrasound is advised for patients with persistent symptoms or diagnostic uncertainty. Diagnostic uncertainty might encompass cases with no identified causative pathogen, for example, a retrospective chart review on patients with epididymo-orchitis (n=118) suggested that men without bacteriuria should be referred to a urologist or scrotal ultrasound if below 50 years of age.^50^ Further large-scale studies could incorporate microbiological data to examine whether bacteriological findings are associated with TC in patients diagnosed with epididymo-orchitis, which may advance the understanding of when to advise scrotal ultrasound in these patients. Moreover, our findings indicate that epididymo-orchitis could be a potential risk factor for TC, which, together with established risk factors (eg, cryptorchidism, genetic factors and familial TC)^4^ may aid in the clinical risk assessment of TC and warrant further exploration. For policymakers, screening all men with epididymo-orchitis for TC would probably offer limited cost-benefit; instead, campaigns prompting awareness of persisting symptoms and self-examination after an epididymo-orchitis may help identify those warranting scrotal ultrasound.

## Conclusions

The association between epididymo-orchitis and TC supports clinicians maintaining heightened awareness for TC in patients with epididymo-orchitis and could provide a foundation for more detailed studies on epididymo-orchitis as a potential TC risk factor. Although a potential link between UTI and TC was identified, the absolute risk was low and cystitis was not associated with TC.

## Supplementary material

10.1136/bmjonc-2026-001078 online supplemental file 1

## Data Availability

Data may be obtained from a third party and are not publicly available.

## References

[1] Znaor A, Skakkebaek NE, Rajpert-De Meyts E (2020). Testicular cancer incidence predictions in Europe 2010-2035: A rising burden despite population ageing. Int J Cancer.

[2] Huyghe E, Matsuda T, Thonneau P (2003). Increasing incidence of testicular cancer worldwide: a review. J Urol.

[3] Huang J, Chan SC, Tin MS (2022). Worldwide Distribution, Risk Factors, and Temporal Trends of Testicular Cancer Incidence and Mortality: A Global Analysis. Eur Urol Oncol.

[4] Singla N, Bagrodia A, Baraban E (2025). Testicular germ cell tumours: A review. JAMA.

[5] Liu S, Semenciw R, Waters C (2000). Clues to the aetiological heterogeneity of testicular seminomas and non-seminomas: time trends and age-period-cohort effects. Int J Epidemiol.

[6] McGlynn KA, Devesa SS, Sigurdson AJ (2003). Trends in the incidence of testicular germ cell tumors in the United States. Cancer.

[7] Stevenson SM, Lowrance WT (2015). Epidemiology and Diagnosis of Testis Cancer. Urol Clin North Am.

[8] Huyghe E, Muller A, Mieusset R (2007). Impact of diagnostic delay in testis cancer: results of a large population-based study. Eur Urol.

[9] Guth M, Coste A, Lefevre M (2023). Testicular germ cell tumour risk by occupation and industry: a French case-control study - TESTIS. Occup Environ Med.

[10] Hermansen M, Hjelmborg J, Thinggaard M (2025). Smoking and testicular cancer: A Danish nationwide cohort study. Cancer Epidemiol.

[11] Brown LM, Pottern LM, Hoover RN (1987). Testicular cancer in young men: the search for causes of the epidemic increase in the United States. J Epidemiol Community Health.

[12] Swerdlow AJ, Huttly SRA, Smith PG (1987). Testicular cancer and antecedent diseases. Br J Cancer.

[13] Garolla A, Vitagliano A, Muscianisi F (2019). Role of Viral Infections in Testicular Cancer Etiology: Evidence From a Systematic Review and Meta-Analysis. Front Endocrinol (Lausanne).

[14] UK Testicular Cancer Study Group (1994). Social, behavioural and medical factors in the aetiology of testicular cancer: results from the UK study. Br J Cancer.

[15] Trabert B, Graubard BI, Erickson RL (2012). Childhood infections, orchitis and testicular germ cell tumours: a report from the STEED study and a meta-analysis of existing data. Br J Cancer.

[16] Shephard EA, Hamilton WT (2018). Selection of men for investigation of possible testicular cancer in primary care: a large case-control study using electronic patient records. Br J Gen Pract.

[17] Trojian TH, Lishnak TS, Heiman D (2009). Epididymitis and orchitis: an overview. Am Fam Physician.

[18] Öztürk Ç, Fleer J, Hoekstra HJ (2015). Delay in Diagnosis of Testicular Cancer; A Need for Awareness Programs. PLoS One.

[19] Kreydin EI, Barrisford GW, Feldman AS (2013). Testicular cancer: what the radiologist needs to know. AJR Am J Roentgenol.

[20] Kao L-T, Lin H-C, Chung S-D (2016). Association between Testicular Cancer and Epididymoorchitis: A Population-Based Case-Control Study. Sci Rep.

[21] Justice ED, Fricker J, Ross JDC (2026). The 2024 European guideline on the management of epididymo‐orchitis. *Acad Dermatol Venereol*.

[22] Wagenlehner FME, Bjerklund Johansen TE, Cai T (2020). Epidemiology, definition and treatment of complicated urinary tract infections. Nat Rev Urol.

[23] Crocetto F, Arcaniolo D, Napolitano L (2021). Impact of Sexual Activity on the Risk of Male Genital Tumors: A Systematic Review of the Literature. Int J Environ Res Public Health.

[24] Ludvigsson JF, Bergman D, Lundgren CI (2025). The healthcare system in Sweden. Eur J Epidemiol.

[25] Barlow L, Westergren K, Holmberg L (2009). The completeness of the Swedish Cancer Register: a sample survey for year 1998. Acta Oncol.

[26] Jansåker F, Li X, Sundquist K (2025). Acute cystitis and subsequent risk of urogenital cancer: a national cohort study from Sweden. *BMJ Public Health*.

[27] Ludvigsson JF, Otterblad-Olausson P, Pettersson BU (2009). The Swedish personal identity number: possibilities and pitfalls in healthcare and medical research. Eur J Epidemiol.

[28] Ludvigsson JF, Andersson E, Ekbom A (2011). External review and validation of the Swedish national inpatient register. BMC Public Health.

[29] Jansåker F, Li X, Knudsen JD (2021). The Effect of Sociodemographic Factors, Parity and Cervical Cancer on Antibiotic Treatment for Uncomplicated Cystitis in Women: A Nationwide Cohort Study. Antibiotics (Basel).

[30] Ludvigsson JF, Almqvist C, Bonamy A-KE (2016). Registers of the Swedish total population and their use in medical research. Eur J Epidemiol.

[31] Ekbom A (2011). The Swedish Multi-generation Register. Methods Mol Biol.

[32] Low M, Almog R, Balicer RD (2018). Infectious disease burden and antibiotic prescribing in primary care in Israel. Ann Clin Microbiol Antimicrob.

[33] Kornfält Isberg H, Hedin K, Melander E (2020). Different antibiotic regimes in men diagnosed with lower urinary tract infection - a retrospective register-based study. Scand J Prim Health Care.

[34] Kornfält Isberg H, Melander E, Hedin K (2019). Uncomplicated urinary tract infections in Swedish primary care; etiology, resistance and treatment. BMC Infect Dis.

[35] Koschel SG, Wong LM (2020). Radical inguinal orchidectomy: the gold standard for initial management of testicular cancer. Transl Androl Urol.

[36] Laguna MP, Pizzocaro G, Klepp O (2001). EAU guidelines on testicular cancer. Eur Urol.

[37] Dahl O (1985). Testicular carcinoma. A curable malignancy. Acta Radiol Oncol.

[38] Naber KG, Bergman B, Bishop MC (2001). EAU Guidelines for the Management of Urinary and Male Genital Tract Infections^1^. Eur Urol.

[39] Ferry S, Burman LG (1987). Urinary tract infection in primary health care in northern Sweden. III. Bacteriology in relation to clinical and epidemiological factors. Scand J Prim Health Care.

[40] Lindgren H (1994). Ultraljud vid akut skrotal smärta. Kan färgdoppler utesluta testistorsion? [English: Ultrasound in acute scrotal pain. Can color Doppler exclude testicular torsion? the Swedish Medical Journal]. Läkartidningen.

[41] Estève J, Benhamou E, Raymond L (1994). Statistical methods in cancer research. Volume IV. Descriptive epidemiology.

[42] Huang C-H, Chou Y-H, Yeh H-W (2019). Risk of Cancer after Lower Urinary Tract Infection: A Population-Based Cohort Study. Int J Environ Res Public Health.

[43] Fan C-Y, Huang W-Y, Lin K-T (2017). Lower Urinary Tract Infection and Subsequent Risk of Prostate Cancer: A Nationwide Population-Based Cohort Study. PLoS One.

[44] Flores-Mireles AL, Walker JN, Caparon M (2015). Urinary tract infections: epidemiology, mechanisms of infection and treatment options. Nat Rev Microbiol.

[45] Uehara T, Takahashi S, Ichihara K (2014). Surgical site infection of scrotal and inguinal lesions after urologic surgery. J Infect Chemother.

[46] Emanuele Liardo RL, Borzì AM, Spatola C (2021). Effects of infections on the pathogenesis of cancer. Indian J Med Res.

[47] Coussens LM, Werb Z (2002). Inflammation and cancer. Nature New Biol.

[48] Wittenberg AF, Tobias T, Rzeszotarski M (2006). Sonography of the acute scrotum: the four T’s of testicular imaging. Curr Probl Diagn Radiol.

[49] Comiter CV, Benson CJ, Capelouto CC (1995). Nonpalpable intratesticular masses detected sonographically. J Urol.

[50] Capet J, Sønsksen J, Bisbjerg R (2018). Is follow-up ultrasound necessary after acute epididymitis? A retrospective analysis from a large university hospital. Scand J Urol.

