# Supplementary material for: Testicular cancer incidence and associations with prior epididymo-orchitis or urinary tract infections: a national cohort study in Sweden, 1964–2018
Source: BMJ Oncol. 2026 Apr 29;5(1):e001078. doi: 10.1136/bmjonc-2026-001078 (PMC13140954; doi:10.1136/bmjonc-2026-001078)
Supplement: online supplemental file 1 [file bmjonc-5-1-s001.docx]

| **Table S1.** Time period and diagnosis codes used to identify the outcome, predictors and comorbidities | | | | | |
| --- | --- | --- | --- | --- | --- |
|  | ICD-10 | ICD-9 | ICD-8 | ICD-7 | ICD-7 |
| **Time period** | 1997–2018 | 1987–1996 | 1969–1986 | 1964–1968 | 1964–2018 |
| **Testicular cancer** (outcome) |  |  |  |  | 178 |
| **Genitourinary infections** (predictors) |  |  |  |  |  |
| Epididymo-orchitis | N45 | 604 | 604 | 614 |  |
| Urinary tract infections (UTI) | N30, N39 | 595, 599 | 595, 599 | 605, 609 |  |
| Cystitis | N30 | 595 | 595 | 605 |  |
| Other UTI | N39 | 599 | 599 | 609 |  |
| **Comorbidities** |  |  |  |  |  |
| Alcoholism | F10, K70 | 291, 303, 571 | 291, 303, 571 | 307, 322, 581 |  |
| Cannabis use | F12 | 304.3, 305.2 |  |  |  |
| COPD | J40–J49 | 490–496 | 490–493 | 500–502 |  |
| Diabetes mellitus | E10–E14 | 250 | 250 | 260 |  |
| HIV | B20–B24, Z21 | 279K, 079J |  |  |  |
| Obesity | E65-E68 | 278 | 277.99 | 287.00, 287.09 |  |
| ICD: International Classification of Diseases (diagnosis codes). COPD: Chronic Obstructive Pulmonary Disease. HIV: human immunodeficiency virus infection. The diagnosis codes N30, 595, and 605 were defined as indicative for Cystitis. The diagnosis codes N39, 599, and 609 were defined as indicative for Unspecified UTI (site not specified). ICD-7 and ICD-8 had not diagnosis codes for HIV or specific diagnosis codes for cannabis use. | | | | | |

| **Table S2.** Testicular cancer by path-histological subtypes (1964–2018, Sweden) | | | |
| --- | --- | --- | --- |
| Subtype | PAD code |  | Cases (%) |
| Seminomas | ‘066’ |  | 6584 (55.3) |
| Non-seminomas | ‘826’ |  | 5009 (42.1) |
|  | Other* |  | 310 (2.6) |
| All |  |  | 11,903 (100) |
| PAD code ‘826’ includes mixed embryonal and teratoid elements. ^*^ Including choriocarcinoma (74 cases), adenocarcinoma (32 cases), adenoid cystic carcinoma (49 cases), liposarcoma (20 cases), unspecified type (57 cases), and other types (78 cases, 1–12 for each specific code). The histologic types of non-germ cell tumors (≈2.3%) were added to non-seminomas in the analysis. | | | |

| **Table S3.** Testicular cancer incidence in the total male population and in those with preceding epididymo-orchitis or urinary tract infections (1964–2018, Sweden) | |
| --- | --- |
| **Total population** |  |
| Study population | 8,382,433 |
| Total follow-up, person-years | 239,890,652 |
| Mean follow-up (±SD), years | 27.93 (±18.95) |
| Total number of individuals diagnosed with testicular cancer | 11,903 |
| Mean age at diagnosis (±SD), years | 36.9 (±13.5) |
| Incidence rate per 100,000 person-years (95% CI) | 5.09 (4.99–5.18) |
|  |  |
| **Individuals diagnosed with epididymo-orchitis or urinary tract infections** |  |
| Individuals diagnosed with epididymo-orchitis (n=89,596) or urinary tract infections (n=294,201) | 383,797 |
| Total follow-up, person-years | 3,012,706 |
| Mean follow-up (±SD), years | 6.85 (±8.31) |
| Total number of individuals diagnosed with subsequent testicular cancer | 522 |
| Mean age at diagnosis (±SD), years | 37.1 (±13.6) |
| Incidence rate per 100,000 person-years (95% CI) | 28.21 (25.89–30.74) |
| CI: Confidence interval; SD: Standard deviation. | |

| **Table S4**. Standardized incidence ratios for testicular cancer diagnosis in men with epididymo-orchitis, by age at the epididymo-orchitis diagnosis (1964–2018, Sweden) | | | | | |
| --- | --- | --- | --- | --- | --- |
|  |  | **Epididymo-orchitis**^*^ | | | |
| Age at diagnosis (yrs) |  | O | SIR | 95% CI | |
| **Seminomas** |  |  |  |  |  |
| <20 |  | 3 | **42.86** | **8.08** | **126.86** |
| 20–29 |  | 37 | **6.73** | **4.73** | **9.28** |
| 30–39 |  | 83 | **5.51** | **4.39** | **6.84** |
| 40–49 |  | 65 | **6.70** | **5.17** | **8.54** |
| 50–59 |  | 23 | **6.12** | **3.87** | **9.19** |
| ≥60 |  | 11 | **3.41** | **1.63** | **6.30** |
| All ages |  | 222 | **5.97** | **5.21** | **6.81** |
| **Non-seminomas** |  |  |  |  |  |
| <20 |  | 11 | **16.42** | **8.15** | **29.48** |
| 20–29 |  | 68 | **6.60** | **5.12** | **8.36** |
| 30–39 |  | 70 | **7.53** | **5.87** | **9.52** |
| 40–49 |  | 16 | **5.00** | **2.85** | **8.14** |
| 50–59 |  | 6 | **5.83** | **2.10** | **12.76** |
| ≥60 |  | 7 | **5.07** | **2.01** | **10.51** |
| All ages |  | 178 | **6.88** | **5.90** | **7.96** |
| **All testicular cancers**^§^ |  |  |  |  |  |
| <20 |  | 14 | **18.92** | **10.31** | **31.83** |
| 20–29 |  | 105 | **6.64** | **5.43** | **8.04** |
| 30–39 |  | 153 | **6.29** | **5.33** | **7.37** |
| 40–49 |  | 81 | **6.28** | **4.99** | **7.81** |
| 50–59 |  | 29 | **6.05** | **4.05** | **8.70** |
| ≥60 |  | 17 | **3.94** | **2.29** | **6.33** |
| All ages |  | 400 | **6.34** | **5.73** | **7.00** |
| Full model, adjusted for period, sociodemographic factors, family history of testicular cancer, and comorbidities. CI: Confidence interval; Cystitis: Lower urinary tract infection; O: Observations; SIR: Standardized incidence ratio; UTI: Urinary tract infection. ^*^ International Classification of Diseases (ICD) diagnosis codes for epididymo-orchitis: N45 (ICD-10), 604 (ICD-8, ICD-9), and 614 (ICD-7). ^§^ ICD diagnosis code for testicular cancer: 178 (ICD-7). | | | | | |

| **Table S5.** Standardized incidence ratios for testicular cancer in men with UTI, by age at the UTI diagnosis (1964–2018) | | | | | | | | | | | | |  |  |  |
| --- | --- | --- | --- | --- | --- | --- | --- | --- | --- | --- | --- | --- | --- | --- | --- |
|  | All UTI^*,§^ | | | |  | Cystitis^*^ | | | |  | UTI, unspecified/site not specified^§^ | | | | |
| Age (years) | O | SIR | 95% CI | |  | O | SIR | 95% CI | |  | O | SIR | | 95% CI | |
| **Seminomas** |  |  |  |  |  |  |  |  |  |  |  |  | |  |  |
| <20 | 0 |  |  |  |  | 0 |  |  |  |  | 0 |  | |  |  |
| 20–29 | 14 | **2.54** | **1.38** | **4.27** |  | 2 | 2.17 | 0.20 | 7.99 |  | 12 | **2.61** | | **1.34** | **4.57** |
| 30–39 | 23 | **2.07** | **1.31** | **3.11** |  | 2 | 0.99 | 0.09 | 3.64 |  | 21 | **2.31** | | **1.42** | **3.53** |
| 40–49 | 10 | 1.15 | 0.52 | 2.18 |  | 3 | 1.67 | 0.31 | 4.93 |  | 7 | 0.99 | | 0.36 | 2.17 |
| 50–59 | 8 | 1.63 | 0.69 | 3.22 |  | 1 | 0.83 | 0.00 | 4.78 |  | 7 | 1.88 | | 0.75 | 3.90 |
| ≥60 | 17 | 1.62 | 0.94 | 2.60 |  | 0 |  |  |  |  | 17 | **2.03** | | **1.18** | **3.25** |
| All ages | 72 | **1.77** | **1.38** | **2.23** |  | 8 | 0.99 | 0.42 | 1.96 |  | 64 | **1.97** | | **1.51** | **2.52** |
| **Non-seminomas** |  |  |  |  |  |  |  |  |  |  |  |  | |  |  |
| <20 | 5 | 2.34 | 0.74 | 5.50 |  | 2 | 5.13 | 0.48 | 18.86 |  | 3 | 1.71 | | 0.32 | 5.07 |
| 20–29 | 21 | **2.00** | **1.24** | **3.07** |  | 4 | 2.21 | 0.57 | 5.71 |  | 17 | **1.96** | | **1.14** | **3.14** |
| 30–39 | 16 | **2.37** | **1.35** | **3.86** |  | 4 | 3.25 | 0.85 | 8.41 |  | 12 | **2.18** | | **1.12** | **3.82** |
| 40–49 | 3 | 1.16 | 0.22 | 3.43 |  | 0 |  |  |  |  | 3 | 1.51 | | 0.28 | 4.46 |
| 50–59 | 2 | 1.46 | 0.14 | 5.37 |  | 0 |  |  |  |  | 2 | 1.92 | | 0.18 | 7.07 |
| ≥60 | 3 | 0.36 | 0.03 | 1.33 |  | 1 | 0.88 | 0.00 | 5.07 |  | 2 | 0.23 | | 0.00 | 1.30 |
| All ages | 50 | **1.70** | **1.26** | **2.25** |  | 11 | **2.01** | **1.00** | **3.60** |  | 39 | **1.63** | | **1.15** | **2.23** |
| **All testicular cancers**^#^ |  |  |  |  |  |  |  |  |  |  |  |  | |  |  |
| <20 | 5 | 2.12 | 0.67 | 4.98 |  | 2 | 4.65 | 0.44 | 17.11 |  | 3 | 1.55 | | 0.29 | 4.60 |
| 20–29 | 35 | **2.19** | **1.52** | **3.04** |  | 6 | 2.20 | 0.79 | 4.82 |  | 29 | **2.18** | | **1.46** | **3.14** |
| 30–39 | 39 | **2.18** | **1.55** | **2.99** |  | 6 | 1.85 | 0.66 | 4.04 |  | 33 | **2.26** | | **1.55** | **3.17** |
| 40–49 | 13 | 1.15 | 0.59 | 2.01 |  | 3 | 1.25 | 0.24 | 3.70 |  | 10 | 1.12 | | 0.51 | 2.13 |
| 50–59 | 10 | 1.59 | 0.76 | 2.93 |  | 1 | 0.65 | 0.00 | 3.75 |  | 9 | 1.89 | | 0.86 | 3.60 |
| ≥60 | 20 | 1.18 | 0.71 | 1.85 |  | 1 | 0.31 | 0.00 | 1.76 |  | 19 | 1.41 | | 0.83 | 2.23 |
| All ages | 122 | **1.74** | **1.44** | **2.08** |  | 19 | 1.40 | 0.84 | 2.19 |  | 103 | **1.82** | | **1.48** | **2.21** |
| Full model, adjusted for period, sociodemographic factors, family history of testicular cancer, and comorbidities. CI: Confidence interval; Cystitis: Lower urinary tract infection; O: Observations; SIR: Standardized incidence ratio; UTI: Urinary tract infection. ^*^ International Classification of Diseases (ICD) diagnosis codes: N30 (ICD-10), 595 (ICD-8, ICD-9), and 605 (ICD-7). ^§^ ICD diagnosis codes: N39 (ICD-10), 599 (ICD-8, ICD-9), and 609 (ICD-7).  ^#^ ICD diagnosis code for testicular cancer: 178 (ICD-7). | | | | | | | | | | | | | | | |

| **Table S6**. Standardized incidence ratios for testicular cancer diagnosis in men with UTI, by time period in relation to the cystitis or UTI (unspecified/site not specified) diagnosis (1964–2018, Sweden) | | | | | | | | | | |
| --- | --- | --- | --- | --- | --- | --- | --- | --- | --- | --- |
|  |  | Cystitis^*^ | | | |  | UTI, unspecified/site not specified^§^ | | | |
| Follow-up times (yrs) |  | O | SIR | 95% CI | |  | O | SIR | 95% CI | |
| **Seminomas** |  |  |  |  |  |  |  |  |  |  |
| The same calendar year |  | 1 | 2.22 | 0.00 | 12.74 |  | 19 | **9.73** | **5.75** | **15.41** |
| Subsequent calendar years |  | 7 | 0.91 | 0.36 | 1.89 |  | 45 | **1.49** | **1.09** | **1.99** |
| 1–4 years |  | 4 | 1.50 | 0.39 | 3.89 |  | 17 | **1.74** | **1.01** | **2.79** |
| ≥5 years |  | 3 | 0.60 | 0.11 | 1.77 |  | 28 | 1.37 | 0.91 | 1.98 |
| All |  | 8 | 0.99 | 0.42 | 1.96 |  | 64 | **1.97** | **1.51** | **2.52** |
| **Non-seminomas** |  |  |  |  |  |  |  |  |  |  |
| The same calendar year |  | 4 | **15.38** | **4.00** | **39.78** |  | 9 | **8.33** | **3.78** | **15.89** |
| Subsequent calendar years |  | 7 | 1.34 | 0.53 | 2.78 |  | 30 | 1.30 | 0.87 | 1.87 |
| 1–4 years |  | 0 |  |  |  |  | 3 | 0.51 | 0.10 | 1.51 |
| ≥5 years |  | 7 | 1.94 | 0.77 | 4.03 |  | 26 | **1.58** | **1.03** | **2.32** |
| All |  | 11 | **2.01** | **1.00** | **3.60** |  | 39 | **1.63** | **1.15** | **2.23** |
| **All testicular cancers**^#^ |  |  |  |  |  |  |  |  |  |  |
| The same calendar year |  | 5 | **7.04** | **2.22** | **16.57** |  | 28 | **9.22** | **6.07** | **13.42** |
| Subsequent calendar years |  | 14 | 1.09 | 0.59 | 1.83 |  | 75 | **1.41** | **1.11** | **1.77** |
| 1–4 years |  | 4 | 0.93 | 0.24 | 2.42 |  | 20 | 1.28 | 0.78 | 1.98 |
| ≥5 years |  | 10 | 1.16 | 0.55 | 2.14 |  | 55 | **1.47** | **1.10** | **1.91** |
| All |  | 19 | 1.40 | 0.84 | 2.19 |  | 103 | **1.82** | **1.48** | **2.21** |
| Full model, adjusted for age, period and sociodemographic factors (educational level, region of residence, and country of origin), family history of testicular cancer; and comorbidities (alcoholism, cannabis use, chronic obstructive pulmonary diseases, diabetes mellitus, human immunodeficiency virus infection, and obesity). CI: Confidence interval; Cystitis: Lower urinary tract infection; O: Observations; SIR: Standardized incidence ratio; UTI: Urinary tract infection. ^*^ International Classification of Diseases (ICD) diagnosis codes for cystitis: N30 (ICD-10), 595 (ICD-8, ICD-9), and 605 (ICD-7). ^§^ ICD diagnosis codes for UTI, unspecified/site not specified: N39 (ICD-10), 599 (ICD-8, ICD-9), and 609 (ICD-7). ^#^ ICD diagnosis code for testicular cancer: 178 (ICD-7). | | | | | | | | | | |
